# Consumer Acceptance of Plant-Based Meat Substitutes: A Narrative Review

**DOI:** 10.3390/foods11091274

**Published:** 2022-04-27

**Authors:** János Szenderák, Dániel Fróna, Mónika Rákos

**Affiliations:** Faculty of Economics and Business, University of Debrecen, 4032 Debrecen, Hungary; frona.daniel@econ.unideb.hu (D.F.); rakos.monika@econ.unideb.hu (M.R.)

**Keywords:** plant-based meat substitutes, alternative protein, sustainable food

## Abstract

The great environmental impact of increasing animal product consumption requires the willingness to reduce or to substitute meat consumption. A possible substitute product, plant-based meat substitute, is made from plants and offers a sensory experience similar to conventional meat. In this narrative review, we focus on the consumer acceptance of plant-based meat alternatives. We searched for peer-reviewed studies in SCOPUS and Web of Science (WoS) up to December 2021. Of all 111 records identified, 28 were eligible, and, thus, included in this narrative review. The results imply that established consumer behavior has complex socio-economic implications for the adoption of plant-based meat substitutes. Plant-based meat was consistently rated more favorably than other meat substitute products, but sensory and nutritional implications still exist. Environmental and health-related factors may contribute to the market spread of plant-based meat substitutes, but these factors alone are not sufficient. Furthermore, so far there is no information available about how the hypothetical measurements used in the studies (such as willingness to pay) will translate to real life consumer behavior. Despite these barriers, there is certainly a great market potential for plant-based meat alternatives, which is expected to be more pronounced in the future, with increasing environmental and health awareness.

## 1. Introduction

In 2018, global agriculture produced around 12% of total greenhouse gas (GHG) emissions (5.82 Gt CO_2_e) without including land-use change and forestry (LUCF), and was the second largest emitter behind the energy sector (78%) [1]. Furthermore, about 21–37% of GHG emissions were attributable to the food system in general [2]. The bulk of emissions were mainly associated with the increasing consumption of animal products, especially meat [3]. Limiting global warming to 1.5 °C requires the reduction in agriculture’s global land footprint. This can be performed by sustainably intensifying agricultural production and/or changing food consumption patterns. This includes the substantial shift of a diet high in ruminant meat towards a higher proportion of plant-based foods and a further reduction in food loss and waste [4,5]. Globally, the demand for animal products has increased due to changing dietary patterns, the rising income of the middle class and population growth [2,6,7]. As incomes rise, especially in the middle class, and people move to cities, diets tend to become more varied and higher in resource-intensive foods, such as meat and dairy [4]. In the developed countries, meat is relatively inexpensive and accessible, but intensive meat production systems have a strong negative impact on the environment [8]. Meat, especially beef, has one of the least efficient calorie conversions of feed to food, thus beef production uses more natural resources (especially land and freshwater) than any other commonly consumed food, and produces more greenhouse gas emissions per unit of protein [9]. Although in countries facing nutrition deficiencies meat is an important source of nutrients, the overconsumption of red and processed meat in the developed countries has been linked to a number of health concerns [8,10]. At the same time, largely plant-based diets were associated with improved cardiovascular health [3,11,12], reductions in the relative risks of type II diabetes, cancer, and corona mortality relative to the average diet, and also with reduced per capita GHG emissions and land clearing [3]. Since livestock production is the single largest driver of habitat loss, a reduction in consumption would also positively impact natural ecosystems and biodiversity [13]. In addition to environmental concerns, meat consumption may even cause moral concerns, resulting in the so-called meat paradox, i.e., the conflict between enjoying meat and concern for animal welfare [14]. The connections between growing population and demand, food security issues, growing energy demand, and environmental concerns have led to a significant competition for natural resources between food, energy, and the environment [15].

Meat consumption is expected to increase over the next few decades, with major effects on the environment, human health and the economics of the food system [7,16,17]. Ruminant meat productivity has increased globally, but not at a sufficient rate (based on the expected increase of 27% per hectare by 2030) and ruminant meat consumption in high-consumption regions is not expected to decline to a sufficient degree by 2030. A further reduction in meat production would be necessary, since this would indirectly contribute to the reduction in deforestation, agricultural production emissions, food loss, and food waste, as well [4]. Due to these tendencies, a substantial dietary shift is required, especially in Western countries [2,18,19] and it has become necessary to reduce meat consumption in particular [4,9,10,13,20].

In addition to an actual reduction in consumption and a shift to different types of diets, plant-based substitutes, laboratory-grown meat and even edible insects have commonly been identified as possible solutions to reduce meat consumption [21,22]. Meat can be replaced with similar products high in protein on meatless days, such as fish, cheese, or eggs [23,24], but these products lack the sensory experience of meat consumption. Plant-based meat substitutes are products made from plants as a substitute for animal-based products [25]. Under the right circumstances, these products certainly have a high potential to became popular, and compared to other substitutes, plant-based meat substitutes were commonly identified as the most similar product cluster to conventional food [26,27]. Plant-based meat substitutes are relatively novel products, while consumption of meat has been present since the beginning of human evolution. Seitan production was first documented from 900 BC to 600 AD, the wheat- and soy-based analogs were commercialized after 1900, while the novel, next generation products were introduced mostly after 1950 [8]. Interest in plant-based alternatives for meat products was known in the early 19th and 20th centuries as well, but the substitution of meat was mainly driven by income reasons. Later, vegetarian and vegan portfolios were introduced, but the taste and texture of these products were not similar enough to meat [28]. Through significant development, plant-based meat substitutes have progressed to novel type of products that are highly effective in mimicking animal-derived meat [8]. Although competitive solutions, such as cultured meat or insect protein production, inevitably suffer from technological scaling, consumers were also reluctant to fully accept these products [6]. Lab-grown beef is the furthest from being currently technologically and commercially feasible and it was among the least desirable meat substitutes [29]. For example, it was reported that in the USA 74% of respondents would choose conventional animal protein over a cultured meat option [30]. Although preferences for alternatives could be strongly correlated [31], plant-based meat substitutes, cultured meat and edible insect protein had their own sensory characteristics [32]; thus the different products may require largely different marketing strategies.

Nowadays, we are already witnessing an unprecedented growth of meat substitutes in the Western market [33,34,35,36]. The market potential was certainly high in the past decade and the year 2020 was a record-breaking year for plant-based related sales and investments globally. Plant-based meat retail sales reached USD 4.2 billion globally with a 24% growth compared to 2019 [35]. Despite these events, the consumer acceptance of novel and unfamiliar foods is still a challenge to market stakeholders [26] and consumer acceptance of meat substitutes are still low [22] or uncertain [2] in several countries. Furthermore, there is limited research on consumer acceptance of and preferences regarding meat alternatives [34,37], and as Goldstein et al. [38] stated, “the portfolio of foods on the market that could realistically be regarded as a plant-based equivalent to beef is narrow”. Overall, there is a concerning lack of research on consumer acceptance of plant-based meat substitutes outside of the West [39] and little research has been performed on how to motivate people to reduce their meat consumption behavior [40]. Improvement in consumer adoption can be crucial, since the success of novel food technologies is strongly dependent on consumer adoption over time [17]. 

The mitigation potential of dietary change depends on consumer choices and dietary preferences, and includes social, cultural, environmental, and traditional factors, as well as income growth [2]. Usually, any change in dietary behaviors due to interventions is slow [7] and drivers of meat consumption are hard to influence through direct policy interventions. As a result, limiting the rise in meat consumption per capita would almost certainly necessitate intervening indirectly on consumer preferences and consumption patterns in order to improve the current linkages between meat consumption and its key causes [16]. The problem is not “all or nothing” in the sense, that the meat industry is an important part of the economy and it provides a livelihood to its stakeholders. Eating modest amounts of meat has health benefits and it can increase food security and dietary quality, especially for poor people. Furthermore, foraging animals can consume foods that are otherwise inedible for humans. Ruminants can graze in marginal areas that are unsuitable for cropping and they can provide benefits via nutrient cycling [3,41]. In addition, sales from animal husbandry can provide income to pay for education and other needs in the poorest regions [42]. Livestock can be necessary to maintain high nature value farmland, depending on the ecological context and land use history. Biodiversity is often positively affected by intermediate levels of disturbance, thus extensively managed and low-input livestock systems can be of high nature value [43]. These factors highlight the importance of meat as a food and add a further layer of complexity. 

Wholesale conversion of a diet away from animal products is not a realistic option, but even incremental steps would be extremely beneficial [44,45], an effective part of which could be to increase the uptake of plant-based meat substitutes. In this review, the factors affecting the consumer acceptance of plant-based meat substitutes were analyzed and systematically summarized. In order to avoid possible biases resulting from the nature of the narrative reviews, a systematic search and assessment were used. We focused only on plant-based meat substitutes and did not discuss the consumption of high-protein plants (such as soy or lentils). Plant-based meat substitutes or alternatives were defined as products that aim to mimic the sensory characteristics of meat but made entirely from plant protein.

## 2. Materials and Methods

### Search Strategy

**Figure A1:**
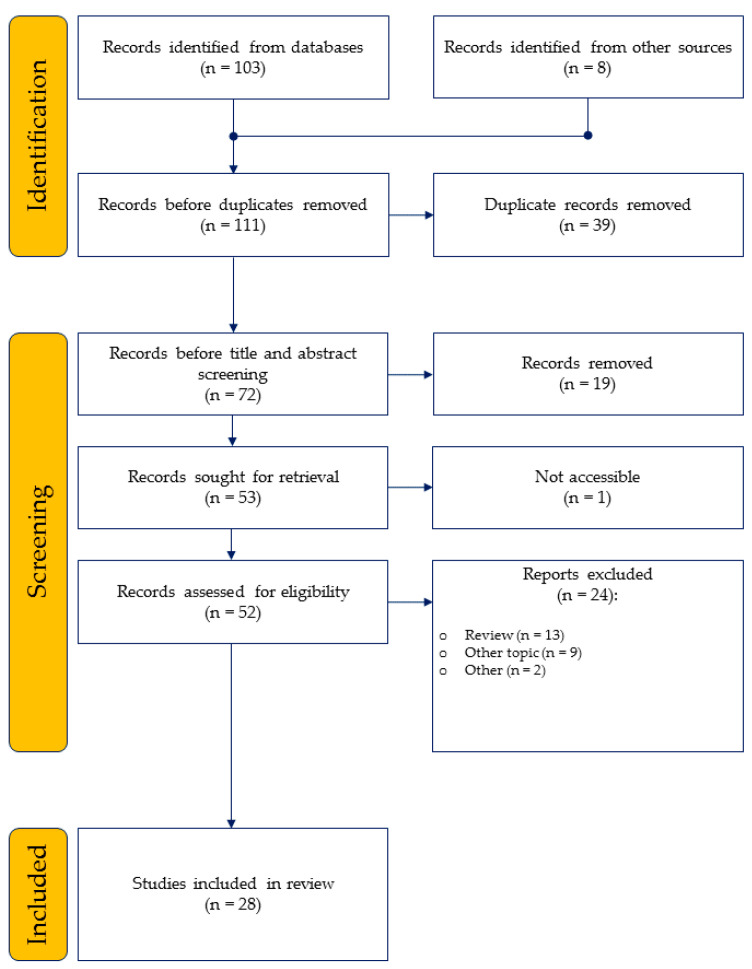
The systematic search process. Source: Authors’ own edition (2022).

A narrative approach was chosen over the commonly used systematic approach, since the aim of this review was rather to provide an interpretive approach for these results and place them in a wider context. However, we followed a rigorous systematic search to avoid subjective selection bias. The steps of the research strategy were the following:

- **Choosing the databases.** We searched the SCOPUS and Web of Science (WoS) databases for peer-reviewed studies up to December 2021. In addition, the International Food Information Council (IFIC) (Source: https://ific.org/, accessed on 3 December 2021) and The European Consumer Organization (BEUC, Bureau Européen des Unions de Consommateurs) (Source: https://www.beuc.eu//, accessed on 3 December 2021) websites were used to search for eight further studies. Furthermore, the recent Smart Protein survey results were also used [34,46]. These studies have been a useful supplement in the area of market segmentation and expected industry trends due to their wide coverage. The inclusion and exclusion criteria are presented in Table 1.
- **Choosing the key words.** The term “plant-based meat” was combined with the keywords “consumer acceptance”, “consumer adoption”, and “consumer purchase”. Furthermore, a different search was made with the term “plant-based burger”, a product which was introduced early on the market and has been in the focus of several studies.
- **Collect the bibliographic data.** The bibliographic data were downloaded in RIS (.ris) format.
- **Pre-processing and screening the articles.** The R software was used to handle the article information, especially the ‘revtools’ package [47], which allowed a flexible interface for title and abstract screening. The ‘revtools’ package supports evidence synthesis projects, aimed at decreasing the time necessary to complete the article screening [48]. The studies were screened based on their titles and abstracts, and the non-related articles were excluded. The process of selection and the details of the studies and reports included can be found in the Supplementary Materials. Although we did not report this review according to the Preferred Reporting Items for Systematic Reviews and Meta-Analyses (PRISMA) [49], the flow diagram presented here was largely based on the PRISMA flow diagram to facilitate comparability (Figure A1).
- **Evaluating the articles and collecting the results based on different themes.** Articles were evaluated based on their final content, which resulted in further exclusion. The final pool of studies consisted of 28 items (21 studies and 7 large-scale survey reports, Table S1, Supplementary Materials). A similar number of results were obtained by Bryant and Barnett [50] and Pakseresht et al. [51] when discussing the case of cultured meat (*n* = 16 and *n* = 43). Further data processing was performed in Tidyverse [52].

## 3. Results and Discussion

### 3.1. Dietary Preferences

The willingness to replace or reduce the consumption of meat can depend to a large extent on current dietary habits. Research to date suggests that the willingness to try plant-based meats is high compared to other alternatives, but the proportion of people who frequently consume meat substitutes is low within the population. We defined the dietary lifestyles in a similar way to the Smart Protein survey [46]. Omnivore individuals consume meat frequently and their diet includes all food groups. Flexitarian individuals consume meat, but they intend to reduce their meat intake and consume higher share of plant-based foods. Pescetarian individuals consume seafood but no other types of meat. Vegetarian individuals do not consume meat but consume other animal-based products, such as eggs or dairy. Finally, vegan individuals do not consume animal-based products at all.

In the USA, more than 92% of respondents mentioned that they ate protein from animal sources, and 72% also ate protein from plant-based sources [53]. The reason behind this was that 50% of the respondents did not follow any specific diet in the USA, while only 11% followed a plant-based diet [54]. Around 66–76% of respondents identified as omnivore and less than 27% as vegetarian, vegan or pescetarian in the USA and for nationalities not specified [29,30,31,55,56,57]. One research study observed much more extreme proportions in the USA, with omnivores above 95% and only 4.6% classified as other [39]. In Europe, 48–74% of respondents identified as omnivores, 20–42% as flexitarian and only 6–14% as vegetarian, vegan or pescetarian, but there were large discrepancies among countries [18,34,46]. Another European survey with the involvement of 11,000 respondents found that 3–7% identified as vegetarian. Furthermore, 6.2% stopped and 35.4% reduced the consumption of red meat, and a further 19.9% intended to do so. Finally, 33.9% of respondents have neither lowered their red meat consumption nor intend to do so [58]. These proportions could be even more pronounced among older consumers (in five European countries), since it was reported that the majority (75–90%) of older respondents did not plan to reduce their meat consumption and around 20–30% did not consume meat substitutes at all. Only 6–18% of respondents planned to increase their consumption [59]. Other research focused on developing countries, such as India, where the share of omnivores was reported to be 80% with almost 15% vegetarian, while the rest were vegan or pescetarian [39]. Other research also found a higher proportion of vegetarians (35%) in India, while the majority (65%) were labeled as non-vegetarian [37]. The proportion of omnivores was above 90% in South Africa, while the proportion of pescetarians, vegans, or vegetarians remained under 10% [17]. Overall, more than two-thirds of consumers were usually identified as omnivores, thus meat played an important role in the average diet. The high proportion of omnivores suggests that the role of meat substitutes may be of particular importance in the transition towards a sustainable diet, as policies to reduce meat consumption are likely to be less effective in these cases.

The role of flexitarians in dietary change is expected to be large, thus greater focus should be placed on them in terms of market research [34]. There was a clear tendency towards meat consumption reduction in Europe, since 46% of consumers reportedly ate less meat compared to a year ago. Around 39% intended to reduce their meat consumption and 25% intended to increase their plant-based meat consumption in the next six months [34,46]. Flexitarian diets might be a more adequate approach to keep a balanced diet and to ensure the adequate intake of essential amino acids and some specific fatty acids compared to a vegan or a more conventional occidental diet. However, the nutritional value of plant-based foods has to be correctly estimated, especially in terms of the bioaccessibility and bioavailability of nutrients [33]. The comparison should be based on extended data collection and improved measurements.

### 3.2. Consumer Preferences and Market Shares

Consumer preferences may predict the effectiveness of different interventions. The frequency, timing, and duration of consumption of meat substitutes are closely related to dietary habits. In the USA, 65% of respondents had consumed plant-based alternatives (defined as products that attempt to mimic the flavor and texture of animal protein but are made with only plant products) in the previous year, while 20% consumed them at least weekly and 22% on a daily basis. Only 22% have not consumed them over the past year and are not interested in trying them in the future [54]. In another survey, only 29% had not tried at least one new type of plant protein in the previous year [30]. Furthermore, 49% had reportedly tried a plant alternative to animal meat [57]. Among the respondents, 18% have purchased plant-based meat alternatives but do not intend to buy them again, 27% have purchased and intend to buy again, while 23% have not purchased but might try them in the future [56]. Another research study found that meat substitutes were purchased less than once a month or at least once a month (but less than once a week) while meat was purchased more than twice a week generally [31]. In Europe, 45–55% of survey respondents never consumed plant-based meat, and 19–21% consumed such products at least once a week in the previous 12 months. Overall, 29–51% reported that they were very or extremely likely to try plant-based meat substitutes if they become widely available and is tasty and affordable and 29–45% of European consumers were more likely to eat plant-based meat substitutes than conventional meat. Overall, 29–47% were likely to purchase it regularly and 18–26% were likely to pay a higher price for it (under the assumption, that it has the same taste and texture as conventional meat). Among the flexitarian consumer group, 53% would eat and 49% would purchase regularly plant-based meat substitutes instead of animal meat (under the assumption, that it has the same taste and texture as conventional meat), but only 25% would pay a higher price for it [34,46]. Another large-scale European survey found that 36.5% of respondents would be willing to replace meat with non-GMO plant-based alternatives, a proportion which would drop to 13.6% in the case of GMO ingredients [58]. Furthermore, around 46% of respondents on average said that they were not willing to reduce red meat consumption, varying from 26% (Italy) to 73% (Greece) [58]. European consumers above 65 years old from five EU countries were usually reluctant to change their diet. The proportion of consumers who did not consume meat substitutes was between 18% and 30%, and only 7–17% intended to increase it, depending on the country [59]. Only 4.0% in Germany and 4.4% in Belgium consumed plant-based meat substitutes frequently, whereas 14.4% and 16.6%, respectively, reported doing so at least sometimes [23]. 

In the USA, only 33% of respondents were very or extremely likely to purchase plant-based meat substitutes during a choice experiment, while in China and India the figure was more than 60% (similar proportions were observed in case of the likelihood of trying) [39]. In India it was also found that around 50% of respondents had strong positive preferences for alternative meat products compared to conventional meat [37]. Furthermore, in a similar choice experiment, respondents opted for the beef burger 69% of the time, when it was available. The plant-based and cultured meat burgers were chosen 27% and 13% of the time, respectively. Although consumers were told that all burgers tasted the same, there was a marked preference for beef burgers (65–75%) and a weak preference for plant-based meat burgers (20–25%) assuming equal prices [31]. In the USA, other research estimated (also via choice experiment) farm-raised beef to be the most preferred product, followed by plant-based alternatives. The different meat alternatives had largely different unconditional predicted market shares, assuming equal prices. The market share of farm-raised beef was between 62% and 72%, while plant-based meat substitutes had choice shares of 15–23%. These shares were only marginally affected by information about the technology or branding [29]. 

Furthermore, omnivores consumed more meat than flexitarians, but flexitarians consumed plant-based protein more frequently than omnivores in Germany [18]. Omnivores evaluated regular meat more favorably while vegans and vegetarians evaluated plant-based meat more favorably than omnivores [55]. Plant-based substitutes were the most favored meat alternatives (>90% of consumers would consume them) in other research as well, while edible insects were least favored (only 26%) for personal consumption [21]. In South Africa, 59.9% of respondents were highly supportive of plant-based meat substitutes, 67.3% were highly likely to try, and 58.8% were highly likely to purchase, but likelihood to pay more was only 31.5%. However, younger generations had slightly higher levels of purchase intention of plant-based meats (60% of the sample compared to 53%) [17]. In Japan, it could be observed that the willingness to try score was generally higher for plant-based meat above cultured meat, insect protein, and 3D-printed foods, and plant-based meat was regarded as the most similar group to conventional food [26]. One research study analyzed meat hybrids in Germany and Belgium, where a fraction of the meat product (e.g., 20% to 50%) was replaced with plant-based proteins. According to the results, consumer preference for plant-based meat was lower compared to pure meat products, but higher compared to vegetarian products that contained no meat at all (the least preferred option) [23].

There was often no information available on what consumers considered healthy, natural or sustainable. Similarly, consumer perceptions of GM ingredients may have interacted closely with perceptions of other attributes of plant-based meat substitutes. Moreover, the results of questions on future purchasing patterns were in some cases entirely speculative and should be treated with caution.

### 3.3. Associations, Meat Attachment (MA) and Food Neophobia (FN)

#### 3.3.1. Associations

The associations made with meat consumption and meat substitutes have been analyzed in various studies. Mostly positive associations have been found with meat, but rather negative associations with meat substitutes [18]. Similarly, consumers also tended to experience positive emotions more often for meat-based burgers compared to plant-based burgers, although negative emotional terms were used less frequently after tasting [32]. In a context analysis used to identify the positive and negative aspects of plant-based meat substitutes in Turkey, negative comments identified these products as “unhealthy” and “tasteless”, while some highlighted that the products were “unusual” or “expensive”. The positive comments mainly highlighted that the products were tasty (“tastes good”) and meat substitutes can “secure food” [22]. In a German sample, associations between females and males differed only for meat, but not for meat substitutes. Females reported concerns about animal welfare and the environment and indicated moderate meat consumption, while males were associated with the positive aspects of meat, such as taste and variety. Regarding meat substitutes, the associated terms were similar; in contrast, meat-related associations differed between females and males [18]. These association were related to the most important personal drivers of (moral or ethical reasons, health or nutrition reasons and environmental friendliness) and barriers to (preference for other sources of protein, aesthetic appeal, too processed, unnatural or artificial products) plant-based meat substitutes consumption [21]. 

#### 3.3.2. Meat Attachment (MA)

Low consumption of plant-based meat substitutes can also be attributed to a strong attachment to meat. The strong dependence of meat consumption on emotional and cognitive dissonance and sociocultural factors is well documented. Meat still has an important social status and is an essential part of a meal [60]. 

Meat attachment (MA) refers to a positive bond with the consumption of meat, comprised of hedonism, affinity, entitlement, and dependence. It was found that, generally, higher meat attachment predicted lower willingness and intention to reduce meat consumption and to follow a more plant-based diet [61]. Some research quantified meat attachment by the meat attachment scale [61], but other research expressed it only indirectly. The evidence was mixed, however, in terms of plant-based meat substitutes. Meat attachment received similar scores in the USA and China, but was lower in India. Higher meat attachment was associated with lower purchase likelihood of plant-based meat substitutes in the USA, while the opposite was found in China and there was no relationship in India. Furthermore, frequent meat consumers were also more likely to consume plant-based meat substitutes in India [39]. Circus and Robison [21] were able to divide their sample into low (39.6%) and high (60.4%) meat attachment consumers. Despite this, 100% of the “low” meat attachment group and 85% of the “high” meat attachment group wanted to consume plant-based substitutes. Other research also found that frequent meat eaters were less likely to purchase plant-based burgers. Not surprisingly, vegetarians exhibited a stronger preference for plant-based rather than cultured meat burgers [31]. Another US-based research found that vegetarians were significantly more likely to choose meat substitutes [29]. Meat was not regarded as an unhealthy food in Germany and Belgium, but rather as an important part of the diet (according to the MA scale) [23]. Similarly, older European meat consumers with high attachment to meat consumed more meat and were less likely to try alternatives [59]. However, there was no connection between the frequency of meat intake and the intention to purchase plant-based meat substitutes in Korea [27] and in South Africa [17]. 

#### 3.3.3. Food Neophobia (FN)

Food neophobia (FN) is defined as the reluctance to eat and/or avoidance of novel foods [62], and can differ according to different factors, such as general familiarity and experience with unusual foods. Plant-based alternatives may have lower food neophobia scores, since they have long been marketed, and thus may be a familiar food to participants [23], although previous bad experience with early meat alternatives, such as tofu, may have reinforced these factors [18]. Furthermore, previously known raw materials used to produce plant-based meats may increase the consumer’s willingness to try newer products. Food neophobia was similar in the USA and China, and slightly lower in India. Food neophobia was negatively associated with the intention to purchase plant-based meat substitutes in the USA, India, and China [39]. This was supported by other research dealing with dish evaluation [55]. Food neophobia had no significant effect in Korea. However, the feelings of unnaturalness concerning plant-based meat substitutes and distrust of biotechnology negatively affected consumers’ purchasing intentions [27]. Similarly, in Belgium and Germany, the food neophobia score was generally low, thus it was not a barrier for the choice of hybrids, although there were negative significant associations with meat attachment and meat substitution [23]. Another research study among the older population in Europe showed that despite the extremely different consumer groups, there was no difference in terms of ‘food fussiness’ (FF) [59], which shares a common etiology with food neophobia [63].

### 3.4. Emotional and Situational Factors

Emotional and situational factors may have an effect on willingness to try. In the USA, most of those who have consumed or would consider trying plant-based meat alternatives consumed (or would have consume) these products at home (75%) or in a restaurant (40%) [54]. The environmental situations that appear to increase the anticipated acceptance of plant-based meat alternatives were similar to those which increase the anticipated acceptance of typical food in Japan. Plant-based meat alternatives were more likely to be eaten at food festivals, restaurants, cafes, and in the home than when at the pub or bar, and with family, friends, and alone than with acquaintances [26]. Eating alone, with friends or with the family on a weekday were perceived equally appropriate occasions to eat meat alternatives for omnivores. However, different situations received low ratings, such as eating meat alternatives for a family Sunday meal, in a restaurant, or for a business lunch [18].

### 3.5. Education

In the USA, higher education tended to be associated with a higher preference for a non-beef alternative [29], higher weekly consumption of plant-based meat substitutes [54,57] and stronger preferences for alternatives [31,39]. Another research study found a negative statistically significant association between education and willingness to buy plant-based meat alternatives in Korea [27], willingness to pay in India [37] and the purchase intention in South Africa [17].

### 3.6. Familiarity

Overall, the results were relatively consistent in that prior knowledge and information positively influence the willingness to try plant-based meat substitutes. In the USA, virtually all respondents were aware of animal protein sources and more than 75–80% consumed them. In contrast, more than 80% of respondents had heard of plant-based alternatives to meat, but less than 40% consumed it occasionally [30]. However, the proportion of respondents very or extremely familiar with plant-based meat substitutes was lower in the USA (19%) compared to China (30%) and India (40%), respectively (however, the samples from China and India were skewed towards more urban-dwelling, well-educated, and high income respondents), and 28–36% of respondents were not familiar at all with plant-based meat substitutes [39]. Another study reported low familiarity among the South African population, since around 17% were highly familiar with plant-based meat substitutes, while 44% were not familiar with it at all [17]. Despite the different proportions, higher familiarity, prior knowledge or curiosity with the products predicted a greater intention to purchase plant-based meat substitutes in the USA, India, and China [39], in Korea [27] and in South Africa also [17]. Previous use of meat substitutes was associated with positive preferences for the choice of meat hybrids, but with differences between product categories in Germany and Belgium [23]. Furthermore, one study observed that frequent meat consumers attached higher importance to the familiarity of food items than eco-friendly consumers [59].

### 3.7. Income and Price Effects

In the US, the likelihood of trying a plant alternative to animal meat increased with income. The most likely consumers (72%) were those with incomes over USD 120,000, while the least likely consumers (35%) were those with incomes less than USD 40,000 [57]. Another survey found that price was rated significantly higher (53% vs. 43%) for animal based-products in the USA [54]. Still, the attachment to the preferred option can be strong; in the US, 45% of respondents would pay more for the preferred animal protein option [30], which highlights its importance in the diet. Another research study found no significant income effect on purchase intention in the USA. Furthermore, 45% of respondents were not at all likely to pay a higher price for plant-based meat substitutes and 18.5% were very or extremely likely to pay a higher price [39]. Other research also found that income had no significant effect on the conditional market shares of meat alternatives in the USA. At the same time, the utility of meat alternatives decreased with increasing price. The mean willingness to pay values were estimated to be 9.17 $/kg (pea protein) and 6.44 $/kg (animal-like proteins produced by yeast), compared to the ‘none’ option. By comparison, the mean willingness to pay for farm-raised beef burgers was above ~22 $/kg in all cases (and was hardly affected by additional information). Providing sustainability information led to a higher mean willingness to pay for the plant-based alternatives while providing information on the branding and technology either reduced the mean willingness to pay or was product dependent [29]. Income had a positive effect on purchase intention in India, where 13.4% of respondents were not at all likely to pay a higher price for plant-based meat substitutes and 52% were very or extremely likely to pay a higher price [39]. At the same time, other research found no significant effect of income on willingness to pay for plant-based meat substitutes in India. However, respondents were willing to pay a premium for plant-based meat substitutes (1.97 $/kg) over the price of conventional meat (aggregating across all four segments), which differed across the consumer segments in terms of value and sign of the relationship [37]. No income effect was found on purchase intention in China, however, where only 9.6% of respondents were not at all likely to pay a higher price for plant-based meat substitutes, and 54% were very or extremely likely to pay a higher price [39]. In Europe, 15% of respondents were not at all likely, while 25% were very or extremely likely to pay a higher price for plant-based meat substitutes than for conventional meat among the flexitarians. Furthermore, 50% of respondents perceived plant-based food products to be too expensive [34]. Similarly in Germany, omnivores rated meat as performing better in terms of price compared to other groups [18]. In the case of European older adults price had no effect on utility, but there was a 10–20% negative willingness to pay for protein-enriched burgers if the burgers were plant-based [59]. Interestingly, in South Africa lower income predicted a higher purchase intention, which could be due to the fact that consumers frame these new meat types as a solution for social equity [17]. Other research has found that price and income had no significant effect on willingness to purchase and price had little importance in the purchasing decision. However, the simulated demand for alternative meat products was sensitive to price, but individuals with a higher preference for plant-based meat substitutes were less sensitive to price [31].

### 3.8. Age

In the USA, the younger generation (18–34 years old) consumed plant-based meat substitutes more frequently and were open to consume it in a variety of locations, while interest in eating plant-based meat substitutes decreased with age. This could be due to the fact that a significantly higher proportion of respondents were following plant-based, vegetarian, or vegan diets compared to other age classes. Furthermore, a significantly higher proportion of younger and middle aged (35–54 years old) respondents consumed plant-based meat alternatives because of environmental/sustainability benefits, texture, or religious or moral reasons. Respondents above 55 years old were more likely to not consume plant-based meat substitutes due to a lack of interest [54]. Furthermore, the younger population had tried plant alternatives to animal meat more often, with those under 45 years of age being the most likely consumers [57]. Other US research also found that older consumers were less likely to choose plant-based meat substitutes relative to younger consumers [29] and that younger consumers had stronger preferences for alternative meat [31]. At the same time, age had no significant effect on willingness-to-buy in Korea [27], in India [37] and in the case of purchase intention in South Africa [17]. Older adults in five EU countries had negative preferences for protein enriched burgers, if the burgers were plant-based, with a strong preference of red meat and poultry above plant-based burgers [59]. 

Other factors emerged in the literature as being of minor importance. The size of the household and having children under 12 had negative effects on the conditional market shares in the USA [29]. If we assume that household size and the number of children increase with age, there may be an interaction effect between similar factors and age.

### 3.9. Environmental Impacts

In a US survey, 71% of consumers were somewhat or very concerned about climate change, and 67% were concerned about the impact of food production on climate change. From those concerned, 71% also mentioned that their concerns at least sometimes impacted food and beverage purchases. Among the food-related activities, 49% of respondents considered the type of food processing and growing to be of the highest importance in terms of their impacts on climate change. The majority of respondents (62%) seek environmentally friendly products in some or all aspects in life, in which food and beverages were the top category (79%) [64]. At the same time, consumers may not be aware of the environmental impact of meat production. Research reported ‘surprisingly low’ consumer awareness of the impact of meat production on the environment [40] and there was significant heterogeneity among the average respondent as to whether meat production was bad for the environment: 16% said they strongly agreed it was and 15% strongly disagreed [31]. In Europe, only slightly over 10% of respondents agreed that their own food habits negatively affected the environment [58].

Despite the confusion, environmental and sustainability concerns are generally regarded as major factors in the increase in plant-based meat substitutes consumption [35]. These results were supported by the review studies in general. In the USA, a significantly higher proportion of respondents rated healthfulness (65% vs. 53%) and environmental sustainability (66% vs. 46%) higher for plant-based meat substitutes [54]. The majority of respondents (47%) rated plant alternatives better in terms of their environmental effect compared to animal meat [64]. Around 66% of respondents believed that an environmentally sustainable diet could include both animal-based and plant-based protein, and a sustainable diet needed to have more plant-based protein (27%) and less animal sourced protein (26%). Most respondents considered planetary health and nutrition as the most important aspects of an environmentally sustainable diet, despite the fact that 40% of respondents was not sure if a ‘sustainable diet’ was the same as an ‘environmentally sustainable diet’ [53]. Less than 20% of the US respondents ranked ‘environmental sustainability’ as the most important factor when considering protein choices [30], and only 35% would buy more environmentally friendly products with the same taste, while 29% would make a choice depending on the cost of the product [64]. This further implies that the ecological reasons (such as biodiversity protection) alone are not sufficient for a dietary shift [6,40].

The importance of the environmental impact of food in purchasing decisions correlated with a stronger preference for both plant-based substitutes and meat burgers. A higher willingness to purchase was reported for those consumers who believed that farming was an important activity for society or meat production was bad for the environment (as originally formulated) [31]. Features of sustainable livestock production positively affected consumers’ purchasing intentions in Korea [27] and environmental factors were also an important predictor of purchase intention in South Africa [17]. Perceived sustainability of plant-based meat substitutes positively affected the likelihood of purchase in India and China [39]. Other research showed that consumers perceived plant-based meat substitutes as effective in addressing global environmental and food security issues, which positively affected the willingness to try the products [21]. One research study showed that sustainability perceptions may depend on the consumer groups questioned. In Germany, omnivores rated meat as performing better in terms of environmental friendliness, while non-meat-eaters and flexitarians perceived meat alternatives as performing better in terms of environmental friendliness [18]. Another study showed that pure meat products blended with plant protein (meat hybrids) received better evaluation than the meat option in terms of environment and animal welfare in Germany and Belgium. Furthermore, environmental labels also had significant positive effects on product preferences [23].

A few neutral results were observed in the literature. There was no significant effect of environmentally conscious behavior on willingness to buy in India [37]. Other research found no significant relationship between the purchasing likelihood and the perceived sustainability of plant-based meat substitutes in the USA [39]. In the case of European older adults, the “eco-friendly” segment attached higher importance to sustainability than frequent meat consumers and had a higher objective knowledge of the environmental impact of food, but there was no difference in preference for different carbon levels for plant-based meat substitutes [59].

### 3.10. Resource Use of Plant-Based Meat Substitutes

One research study used life cycle assessment (LCA) to measure the environmental impact of a novel vegetal protein source in the mean US diet where it replaces ground beef, and in vegetarian and vegan diets where it substitutes for plant protein sources. It was estimated that the vegetarian and vegan diets reduced per-capita food-borne GHG emissions (by 32% and 67%, respectively), blue water use (by 70% and 75%, respectively) and land occupation (by 70% and 79%, respectively) relative to the mean US diet. In the vegetarian diets, higher dependence on dairy as a protein and fat source increased the environmental impact. The environmental performance of the mean US diet was improved by all three metrics since the plant-based burger was an ecologically leaner protein option in the mean US diet. The substitution of 10%, 25%, and 50% of ground beef with plant-based burgers at the national scale resulted in reductions in annual US dietary GHG emissions, water consumption and land occupation. For vegetarian and vegan diets, the results were more complex, since a few measures increased (GHG emissions increased for the vegetarian and vegan diets by 3–17% and 8–38%, respectively). The increased GHG emissions were due to the energy input requirements of the plant-based burger, which were higher than soy and nut-based protein sources due to production processes and the inclusion of leghemoglobin. Although plant-based burgers had significantly less impact on the environment than beef, they were similar to other animal proteins and higher than other plant-based alternatives [38]. Another research study compared the environmental performance of burger patties made from extruded meat substitutes, produced with different extrusion technologies. Beef burger patties had the highest overall environmental impact in most categories, except for terrestrial and freshwater ecotoxicity, urban land occupation, natural land transformation, and metal depletion. Overall, plant-based patties were more environmentally sustainable than meat burger patties. Beef burger patties had almost 95% higher impact in every endpoint category regardless of the extrusion technology used. Hence, plant-based patties not only have a considerably low environmental impact, but also score better in every endpoint category. Meat-based burger patties have an at least five times higher total environmental impact than plant-based burger patties (despite the raw material and extrusion technology used). Although chicken and pork burger patties also had high relative impacts in most midpoint impact categories, vegetable protein patties were not “environmentally friendly” in every midpoint impact category. Furthermore, the overall impact was dependent on the extrusion technology used as well [65]. The environmental performances can be very sensitive to the changes of functional unit (unit of mass or volume). For example, chicken meat and insect-based meat substitutes may perform better than the other alternatives in case of alternative functional units. This reflects the heterogeneity of the results. The assessment of environmental performance therefore requires several types of sensitivity testing [66]. 

In addition, a trade-off between consumer habits, preferences and environmental impacts may exist. It is important to clarify the impact of food on the environment, since for some choices, consumers may mistakenly believe that they have chosen the food with the lowest environmental impact. Similar findings were presented by Broeckhoven, Verbeke, Tur-Cardona, Speelman, and Hung [59] and, as noted, skepticism towards or disbelief in far-reaching environmental claims can lead to sub-optimal decisions as well.

### 3.11. Gender

In a US survey, a statistically significantly higher proportion of men than women consumed plant-based meat substitutes daily [54], and men (53%) were more likely to be consumers than women (44%) [57]. Furthermore, men were more likely to choose the non-beef alternatives [29]. However, other research analyzed found no difference in the intention to purchase plant-based meat substitutes in the USA [39]. Other studies have found no significant difference in terms of willingness to pay or purchase intention in India [37,39], willingness to buy in Korea [27], or purchase intention in South Africa [17]. These results were also supported by another hypothetical choice experiment [31]. At the same time, women were more likely than men to buy plant-based meat substitutes in China [39]. Among older European consumers, more females were identified as eco-friendly consumers compared to the ‘meat lovers’ cluster (55% vs. 42%), where the acceptance of plant-based protein was higher [59].

### 3.12. Religious and Political Views

Religious or moral reasons emerged as the least common reasons (8%) for consuming plant-based meat alternatives in the USA [54]. Despite this, other research found significant differences in terms of political views, and more liberal people reported a higher intention to purchase plant-based meat substitutes in the USA [39]. Other research found that respondents who identified as politically liberal were also more likely to purchase alternative meat [31]. At the same time, no differences were found in terms of political attitudes in India [37] and in South Africa [17]. However, there were significant differences in terms of willingness to pay according to religion in India [37], which was not observed in other studies.

### 3.13. Sensory Characteristics

Taste usually emerged as the most important factor in the USA [30], and regardless of the source of protein, more than 70% of consumers choose protein mainly based on taste [53]. Despite the continuous development of plant-based meat substitute products, taste was rated significantly higher for animal-based products in the USA [54]. Similarly, meat was generally regarded as tastier compared to substitute products by more than 60% of respondents in Germany and in Belgium [23]. In the USA, most respondents (41%) were motivated to try plant alternatives to conventional meat because they liked to try new foods. Interestingly, most respondents (53%) in a US survey liked the taste most when considering preparing/eating the plant alternative to conventional meat. The criticism of plant-based alternatives to meat covered mostly the texture, or the respondent simply did not like it all (there was nothing they liked about it). Among those who did not consume plant alternatives to animal meat, anticipation of not liking the taste was among the top reasons given (31%) [57]. Taste was also the leading factor for European consumers (40%) when choosing plant-based food products [46]. Taste is usually a general requirement and the importance attached to sensory aspects can be very similar even among consumer segments with largely different consumption habits [59]. One research study found that product characteristics (appeal, excitement, taste, necessity, and disgust) had country dependent effects on purchase intention in the USA, China, and India [39]. In South Africa, not only taste, but all sensory characteristics were regarded as important and there was no large difference in the importance of characteristics of plant-based meat substitutes. However, beef and poultry were the most appealing product types, followed by pork and mutton [17]. Taste, texture, and the protein content of meat were rated higher by omnivores, and very similarly by flexitarians, whereas non-meat-eaters evaluated meat alternatives as performing better regarding all rated aspects except price in Germany. Despite the importance of taste, there was only a weak preference for several meat alternatives to taste like meat, with no difference between omnivores and flexitarians [18]. Other research seems to support the idea that taste has only a small effect on the probability of purchasing plant-based meat substitutes [31]. The success of consumers’ perception of a product as similar to meat also depends on the type of product to which it is compared. For example, the classic steak was always perceived as the most festive, healthy, masculine, expensive, tasty, natural, filling, and protein rich among the tested foods. Furthermore, meat products were rated as tastier compared to meat alternatives, but chicken nuggets, vegetarian nuggets, and vegetarian sausage often received similar ratings [18].

Experiments under blind (only tasting), expected (information provided but no tasting) and informed (information provided with tasting) conditions seems to prove that meat products are strongly preferred over plant-based alternatives in terms of sensory characteristics. Under the blind condition, only the meat burger was strongly preferred compared to the plant-based burgers. Compared to the meat burger, plant-based burgers had an off-flavor; they were perceived as less juicy, dry, and granular under the blind condition. Perceived quality and nutritiousness improved during the expected and informed conditions and the plant-based burgers were perceived as more nutritious compared to the meat-based burger during the informed condition. However, the information on the composition barely influenced sensory perception. [32]. In this regard, sensory data would be key to understanding the physiochemical characteristics of novel plant proteins [67] in order to support sensory developments.

### 3.14. Nutritional Profile

Plant-based meat substitutes are able to only partially mimic meat, thus sensory and nutritional implications still exist. Animal-based proteins provide all of the essential amino acids in ratios needed for normal body function, and are thus considered “complete” sources. Plant-based protein sources lack the proper amounts of one or more of the essential amino acids and are considered “incomplete” [28]. Research showed that both plant-based and meat-based burgers available in the supermarkets of the EU have similar protein profiles and saturated fat content. The most abundant minerals were Ca, K, Mg, Na, P, and S in both plant-based and meat-based burgers. Na, S, and Si content was similar, with Zn being less abundant in plant-based burgers. There were no differences found in total protein and fat content (the latter due to the use of coconut oil as an ingredient), but the protein profile showed that 5 out of the 18 amino acids differed significantly and plant-based burgers had a much lower cholesterol content (a median of 3.98 mg/100 g of the raw product compared to 50.60 mg/100 mg) [33]. Other research supported the idea that the available plant-based burgers have macronutrient profiles similar to 80% lean ground beef burgers, especially with regard to their fat and saturated fat contents. Although the sodium levels were higher in plant-based burgers, the bioavailability of protein, calcium, and iron were significantly lower. The calorie, protein, and total fat contents were relatively similar, yet the 95% lean ground beef burger had a lower calorie and a higher protein content. At the same time, the quality and bioavailability of the protein source varied. Overall, animal-based protein sources contained a more complex dietary profile of vitamins and minerals, whereas plant-based burgers had higher fiber and calcium contents due to added ingredients [28]. Different factors can impair the sensory experience of plant-based meat substitutes. The color of the products may fade out due to light or oxygen exposure, leading to an unappetizing appearance. Off-flavors from lipid oxidation of unsaturated fatty acids in plant protein ingredients and products may lead to an undesirable taste. Due to the reduced saturated fat content, it is very challenging to recreate the texture characteristics of muscle protein, such as fibrous structure, tenderness, and juiciness [67].

Plant-based meat substitutes lie on a spectrum. At one end, there are types of plant-based meat substitutes which are perceived as more natural, containing less-processed proteins, but with a less realistic sensory experience of consuming meat. At the other end of the spectrum there are sensory counterparts that necessitate a complete transformation of source proteins, which are regarded as highly processed and may come at the expense of some nutritious ingredients [8]. The market for ultra-processed plant-based substitutes for meat and dairy is expanding [68]. It is important to note that additives are necessary for highly processed products. Furthermore, highly processed products may have higher environmental impacts too.

## 4. Miscellaneous Topics

### 4.1. Health Perception

Plant-based diets were shown to have a positive impact on health [3,10]; thus health attributes may have a key role in the uptake of such products. Among European consumers, 58% associated the consumption of high amounts of meat with serious health problems. Around 51% of people would reduce their meat consumption if their doctor recommended doing so [46], which implied the presence of strong, health related perceptions.

The majority of the respondents (39%) consumed plant-based alternatives because of healthfulness in the USA, but 34% of respondents indicated the importance of high-quality protein or the good taste (‘like the taste’) [54]. Healthiness (34%) and freshness (29%) were among the top reasons when choosing plant-based food products in Europe as well [46]. The word ‘healthy’ was strongly associated with plant-based alternatives; even the macro-nutrient content of plant-based burgers and traditional beef burgers is similar [29]. Health was also among the strongest motivating factors in purchase intention in South Africa [17], and pure meat products were perceived as the less healthy option compared to meat hybrids and vegetarian alternatives in Germany and Belgium [23]. Health aspects may differ among older consumers according to the different consumer segments. For example, the “eco-friendly” segment attached higher importance to health attributes compared to frequent meat consumers [59]. 

One research study showed that plant-based products were perceived as healthier and more eco-friendly when described as “plant-based” rather than “meat alternative”. However, no effects were found on perceptions of ethicality, perceptions of enjoyment and predicted satiety. Furthermore, participants were more likely to try the product described as plant-based but predicted eating greater quantities of the product described as a meat alternative. There was no evidence that product descriptor and packaging color impacted perceptions of healthiness, expected enjoyment, ethicality, nor predicted quantity consumed [69]. 

Consumers were shown to be interested in the nutritional value of the products. For example, in the USA, nutrition facts about products were more influential than a list of ingredients when choosing a product [57]. Plant-based alternatives were generally regarded as healthier than animal meat, but nutrition facts and lists of ingredients changed consumer shares [56,57]. In terms of information, vitamin and mineral information were the most influential for those who say the plant alternative is healthier than ground beef. However, when a ground beef label was presented with more vitamins and minerals, the role of cholesterol and protein became the most important. Finally, in those cases when the consumer considered the plant-based alternative to be less healthy, sodium content was the most influential factor [56].

In addition to food packaging, health websites were the most common sources respondents would look to for information about plant-based meat substitutes in the USA. Around 50% of respondents would use the nutrition facts label or the ingredient list as information sources when choosing a plant-based meat substitute [54]. Similarly, European consumers reportedly trusted health/nutrition society websites (52%) and search engines (50%) the most to get information about plant-based foods products, so it was not surprising that search engines (58%), health/nutrition society websites (46%), and online videos (41%) were used in most cases to do so [46]. However, social media was not perceived as a very trusted source [34]

### 4.2. Labeling

Similarly to traditional food products, labeling policy may affect the product choice [70,71], and different label attributes could interact with plant-based product attributes. The importance of products’ naturalness have appeared relatively frequently for plant-based meat substitutes. Consumers’ perceptions of (conventional) food naturalness is a key to consumer acceptance, especially the food’s origin, the technology and ingredients which have been used and the properties of the final product [72]. This was supported in the case of both animal protein and plant-based meat substitutes in the USA, since the ‘all natural’ label was also the most appealing label term when buying these products, regardless of the type [56]. The labels “natural”, “organic”, and “locally produced” were perceived as conveying a positive impact on climate change with around a 42% share [64], while the most important plant protein labels in the USA were ‘good source of protein’, ‘natural’, and ‘organic’ [30]. Similarly, one research study also showed that those who believe food should be natural were more likely to purchase plant-based burgers [31]. More than half of European consumers trusted that plant-based protein foods were safe (51%) and accurately labelled (50%). Furthermore, 49% of respondents considered organic label plant-based food products to be important [46]. Organic and local origin labels had a positive effect on product choice in the case of meat hybrids. The same results were recorded partly for national and applied health labels as well, the effect varying depending on the products and their attributes [23]. At the same time, older European adults not only had negative willingness to pay (WTP) when protein-enriched burgers were plant-based, but the same applied to carbon-friendly labels. Beef and poultry burgers were usually preferred with carbon-friendly labels [59]. Accurate labeling can be crucial, since a lack of knowledge and a difficulty in identifying sustainable food options were among the main barriers to sustainable eating in Europe [58].

There was an overlap between the appealing animal-based and plant-based product label terms. In the USA, environmentally sustainable animal protein was associated with the labels ‘no added hormones’ (50%), ‘grass fed animals’ (40%), and ‘locally raised’ (32%) animals [53]. Another survey showed that the most important animal protein labels for consumers were ‘no antibiotics’, ‘natural’, and ‘no added hormones’ (with a proportion of 22–23%) [30]. One study showed that information had only a small impact on consumer choice, which varied depending on the types of benefits communicated to US consumers [29]. In addition, other research found that similar attributes (such as opinions about other technologies and organic food, and favorable views towards genetically modified products, processed or locally produced food) did not have a significant effect on product preferences, although the effect differed strongly between consumers [31]. One of the key issues with labelling is how accurately it conveys information to the consumer. This is particularly important as plant-based products are not necessarily more natural, healthier, or sustainable than their conventional counterparts.

### 4.3. Additional Factors of Consumer Acceptance

#### 4.3.1. The Economics of Production

In addition to taste and accessibility, price has a key importance in driving interest in alternative proteins [34]. Plant-based foods were often more expensive than animal-based products [8,34,73], despite the fact they were often far cheaper to produce due to the less costly raw inputs (unprocessed plant proteins) [8,34]. Higher retail prices were due to the costs associated with post-processing, production scale and supply chains [8]. The relatively high price of plant-based meat products was consistently highlighted as a barrier to trial [35]; furthermore, the significant premium consumers had to pay varied strongly by country [73]. In Europe, price (52%) was highlighted as the main barrier to eating more plant-based foods, beside lack of information (45%) and lack of choice when eating out (41%) [46], while price was the second most important barrier after taste in the USA, as well [30]. In addition, high price was the main barrier to sustainable eating in Europe and only one in five consumers were willing to spend more money on sustainable food [58]. Furthermore, 47% of European consumers considered plant-based meat as too expensive (but also considered eating meat at every meal as expensive) [46]. Other research showed that a USD 1.00 increase in the price of simulated meat (from the baseline of USD 4.00), resulted in a 6% hypothetical increase in the market share of beef [31]. This means that meat substitutes could replace meat successfully only if they resemble highly processed meat products in taste and texture and at the same time, are offered at competitive prices [18]. The reduction in price could contribute to the transition to sustainable food systems and are in line with the EU’s Farm to Fork Strategy [74].

In order to became competitive, the product price should be decreased. In the past few years, several plant-based meat substitute producers have been able to lower their prices and move closer to price parity, which is an important step towards consumer adoption of plant-based meat substitutes [35]. This gap may decrease if producers are able to scale up production, achieve economies of scale, and seek price parity. Closing the price gap would likely increase the purchase intention for most consumers [73]. With increasing affordability, alternative protein may unlock the omnivore market and can compete with conventional products [73].

In terms of ingredients, most of the raw materials have been approved for human consumption previously, thus the regulatory environment is not expected to be a barrier (except for novel ingredients) [8]. Furthermore, genetically modified ingredients can create dilemmas for market stakeholders [58]. However, experimentation is expensive, while machinery is highly capital intensive, which remains a barrier in reducing costs. A significant economic bottleneck is the creation of a proper texture for the industry. In addition, protein crops should be optimized for use in alternative-protein products and protein extraction must be further improved as well [10]. It is vital to reduce the cost and complexity of additives. New flavoring and texturizing substances will be required to achieve parity. Consumers seek foods that are fully free of animal products, which necessitates the development of alternatives for regularly used binding agents, such as gelatin and egg whites. However, because foods must be “natural,” substances like methylcellulose, a chemical utilized as a binding agent in a variety of sectors, are becoming less popular [10]. This indicates that industry may have better options to replace processed meat products in taste and texture, since different factors (such as spices) are responsible for consumer experience, not only meat [18,24]. Generally, highly structured products and large cuts of meat (such as brisket or steak) are the least likely to be replaced at parity by 2035 [10].

#### 4.3.2. Competing Products

Other competitors, such as cell-based meat production, are not yet commercially feasible without further increases in production scale and reductions in culture medium-cost. Plant-based sources not only had the highest (41–56%) interest [54], but were among the most trusted [46] and most accepted alternative proteins [58]. Cultured meat prompted the least interest among the meat alternatives (14–17%) [54] and it is expected to be the last to reach price parity [36]. At the same time, the estimated uncertainty around cultured meat preferences may be largely due to the significant heterogeneity in preferences in the population [29]. It is worth noting that cultured meat can be regarded as ‘real’ (i.e., animal-based) meat, thus it can be attractive to frequent meat consumers. Insect protein is faced with similar barriers, such as consumers’ willingness-to-try [6,32], its cost-effectiveness, reliable production, food safety and regulatory issues [75]. However, around 2 billion people consume insects as part of their diet and around 2000 insect species harvested from nature have been used as food. In addition, insect protein can be considered nutritious and has various environmental benefits compared to conventional animal protein [76]. Still, a European survey found that around 77% of respondents would not be willing to replace meat with insects or with cultured meat (67%) [58]. In addition, animal welfare and ethical issues require further research [77].

#### 4.3.3. Problems of Naming

There are ongoing debates surrounding labeling laws regarding what products can be characterized as meat [8,35,58]. On the consumer side, a few research studies have reported results in relation to naming. For example, 49% of respondents indicated that ‘chicken patty’ is a rather inaccurate and misleading term for plant-based meat alternatives, while around 70% of respondents found the ‘100% plant-based patty’ and ‘plant-based patty’ to be rather accurate and clear terms in a US survey [54]. In another research study, more than 70% of the respondents indicated that the United States Department of Agriculture (USDA) and the United States Food and Drug Administration (FDA) should prohibit the word ‘beef’ on the packaging of meat alternatives and 81% supported the use of the word ‘beef’ only for cattle-derived products [29]. However, as other research highlighted, 42% of respondents were not concerned by labels if the products were clearly labelled as vegetarian/vegan, and a further 26% had no problem at all with meat-like names for plant products in Europe [58]. Regulatory bodies need to establish optimal naming conventions, in line with market stakeholders, that accurately inform consumers but also convey the environmental and health benefits of plant-based meat substitutes.

#### 4.3.4. Future Prospects and Research Directions

Plant-based meat substitutes appear to fit more in the “niche” category [29], but in general, alternative proteins have morphed from a niche product to a mainstream phenomenon [34,35,36]. There is clearly future market potential for plant-based meat substitutes [29], especially in China and India [39]. Other regions may also emerge; for example, South Africa based on its great interest in plant-based meat substitutes [17]. North America and Europe can be considered the most mature markets for alternative proteins, although the Asia-Pacific region may provide the largest opportunities. In this region, population growth, rising incomes and increasing protein consumption drives the market [10,78]. In developing countries, other aspects may emerge as well. For example, local food security was an important predictor of purchase intention in South Africa [17], which can be taken into account in future research. Still, the future for plant-based meat alternatives remains highly uncertain, since part of the current demand may only be a result of novelty and not a long-term trend [29]. Furthermore, the speculative nature of the estimates has to be taken into account, since the superior performance of plant-based foods largely results from a hypothetical large-scale adoption [38].

Future research should cover how plant-based meat substitutes may implement product and marketing strategies from the milk and other dairy alternative market, since these products are the most widely used alternative-protein products [10]. Furthermore, there remains the question of how much consumer acceptance can be hindered by previous bad experiences/memories of “classical” meat substitutes (such as tofu) [18], or how much it can be facilitated by blended products, such as meat hybrids [23]. Other research has highlighted that it would be important to screen new protein sources that may mimic meat without excessive human manipulation, while providing a balanced amino acid profile with the complementary addition of multiple plant-based proteins [8]. To reinforce the adoption of meat analogue products, further research should also focus on the intervention effectiveness of reducing meat consumption. The effectiveness of these interventions was shown to depend on similar factors that increase consumer acceptance of meat analogue products [79].

### 4.4. Limitations of the Research

The following limitations were identified in this research:

- The possible pool of studies may vary, based on the different search terms. A large body of literature is related to the different aspects of plant-based diets, which may contain useful information, but was missed due to the restrictions on the search terms;
- Only English language studies were included, which further limits the scope of the results, although this source of bias is considered marginal;
- Studies used definitions differently, which made the synthesis process more difficult. Differences arising from the different definitions may introduce some confusion into the results. Several studies used willingness to pay, willingness to purchase, willingness to try, willingness to consume, purchase intention and similar terms, which we attempted to summarize uniformly;
- Methodological constructs aimed to measure physiological factors or perceptions should be handled with care, while the possibly wide uncertainty should be acknowledged in these studies and during the interpretation;
- Questions aiming to measure the willingness to purchase or willingness to try of plant-based meat are hypothetical by nature, thus large-scale adoption remains speculative;
- Finally, some interventions appear to reinforce each other, thus the market effect may differ from the effects recognized in the studies included.

## 5. Conclusions

Plant-based meat substitutes can play a crucial role in reducing the burden on the environment and, thus, in the fight against climate change. More than two-thirds of consumers were usually identified as omnivores, thus meat played an important role in the average diet. One-third (or less) of consumers were identified as vegetarian, vegan, or pescetarians. Omnivores were characterized by higher meat consumption, and frequent meat eaters were less likely to choose plant-based substitutes. Food neophobia and regulatory issues are not expected to be a barrier to market uptake, since plant-based meat alternative ingredients are previously consumed raw materials. However, attachment to meat can be high, not only physically but also psychologically, which was highlighted in the consumption trends and in the associations related to conventional meat and plant-based meat substitutes. Overall, the results were relatively consistent in that prior positive experience and knowledge predicted higher willingness to try. It was likely that consumers with higher incomes were more likely to choose plant-based alternatives, and specific groups were willing to pay a higher price for such products. However, plant-based meat substitutes are still priced at a premium compared to conventional meat. In addition to taste and texture, a competitive price is crucial in market penetration. Environmental factors played a decisive role, but environmental reasons alone are not likely to be sufficient to cause a large-scale dietary shift. A separate issue is what consumers consider to be sustainable or healthy. Based on the results, it was clear that the environmental impact of plant-based meat substitutes was substantially lower compared to the average diet. Health considerations were also important, although, highly processed plant-based meat substitutes are not necessarily healthier than conventional meat dishes. Taste and texture are critical, but it is also advantageous that there is no significant difference in terms of texture between plant-based and conventional meats. Increased emphasis on environmental and health related arguments could help to promote the uptake of these products. However, this also requires a competitive price. One of the greatest challenge seems to be related to the hypothetical nature of the measurements used in the studies (willingness to purchase or willingness to pay for example). So far, there is no information about how these measurements will translate to real life consumer behavior. Despite these barriers, there is certainly a great market potential for plant-based meat alternatives, which is expected to be more pronounced in the future with increasing environmental and health awareness. Finally, it is worth emphasizing that what is required is not a complete dietary change, but a transition to sustainable food consumption in incremental steps.

## Figures and Tables

**Table 1:** Inclusion and exclusion criteria used during the search.

Search Criteria	Web of Science (WoS)	Scopus
**Keywords**	(“plant-based meat *”) AND (consumer * accept *, consumer * adopt *, consumer * purch *) (“plant-based burger *”)	
**Search type**	All Fields	Article title, abstract and keywords
**Year**	all available	
**Languages**	English	English
**Inclusion criteria**	Focus on the aspects of consumer acceptance of plant-based meat Studies published in peer-reviewed journals or related organizational websites Studies written in English Empirical studies or reviews with strong relevance	
**Exclusion criteria**	No discussion of consumer behavior Conference papers, abstracts, and educational papers Focus on purely technological innovations	

Source: authors’ own collection (2021). *: The asterisk is a wildcard.

## Data Availability

Not applicable.

## Supplementary Materials

The following supporting information can be downloaded at: https://www.mdpi.com/article/10.3390/foods11091274/s1. Table S1: Main features of the studies reviewed.
