# Supplementary material for: Consumer Acceptance of Plant-Based Meat Substitutes: A Narrative Review
Source: Foods. 2022 Apr 27;11(9):1274. doi: 10.3390/foods11091274 (PMC9102955; doi:10.3390/foods11091274)
Supplement: Supplementary file 1 [file foods-11-01274-s001.zip › foods-1693522-suppl.pdf]

Table S1. Main features of the studies reviewed.

	Authors	Methods	Sample	Focus (with the original description)	Region
1	Arora et al. (2020) [37]	discrete choice experiment	394	conventional meat, plant-based meat, cell-based meat, and chickpeas	India
2	BEUC (2020) [58]	survey	11 000	sustainable food, which included plant-based meat	Europe (11 countries)
3	Broeckhoven et al. (2021) [59]	discrete choice experiment	2 500	protein-enriched burgers	Europe (5 countries)
4	Bryant et al. (2019) [39]	ANOVA and linear regression by country	3 030	plant-based meat, cultured meat	USA, India, China
5	Circus and Robison (2019) [21]	interviews and online survey	7 interview, 139 surveys	plant-based meat, cultured meat, edible insects	not explicitly stated
6	De Marchi et al. (2021) [33]	nutritional profiling	-	meat-based burgers and plant-based burgers	-
7	Edge and Garrett (2020) [28]	nutritional profiling	-	plant-based burgers	-
8	Fiorentini et al. (2020) [67]	review	-	plant-based meat analogs and meat extenders	-
9	Goldstein et al. (2017) [38]	life cycle assessment (LCA)	-	plant-based burger	USA
10	Hwang et al. (2020) [27]	linear regression	504	plant-based meat, cultured meat	Korea
11	IFIC (2019) [53]	online interviews	1 000	plant alternatives	USA
12	IFIC (2020b) [56]	online interviews	1 000	plant alternatives	USA
13	IFIC (2020c) [57]	online interviews	1 000	plant alternatives	USA
14	IFIC (2021a) [54]	online interviews	1 001	plant-based meat alternatives	USA
15	IFIC (2021b) [30]	online interviews	1 009	protein	USA
16	Krings (2022) [55]	ANOVA	655	regular meat, clean meat, plant-based meat	Not stated
17	Michel et al. (2021) [18]	ANOVA	1 039	general meat alternatives	Germany
18	Motoki et al. (2022) [26]	ANOVA and linear regression	117, 108 and 120	plant-based meat, cultured meat, edible insects, 3D printed foods	Japan
19	Profeta et al. (2021) [23]	discrete choice experiment	500 and 501	meathybrids	Germany, Belgium
20	Rubio et al. (2020) [8]	review	-	plant-based and cell-based meat	-
21	Saerens et al. (2021) [65]	life cycle assessment (LCA)	-	plant-based burger patties from different ingredients with different technologies	-
22	Schouteten et al. (2016) [32]	emotional and sensory profiles of products, ANOVA, t-test	97	meat-based, plant-based and insect-based burgers	not explicitly stated
23	Slade (2018) [31]	discrete choice experiment	533	plant-based meat, cultured meat burger, conventional meat burger	not explicitly stated
24	Smart Protein (2021a, 2021b) [34,46]	online survey	7 590	plant-based food	Europe (10 countries)
25	Sucapane et al. (2021) [69]	ANOVA, t-test	149, 182 and 368	plant-based meat	USA, Canada
26	Szejda et al., 2021 [17]	linear regression	959	plant-based meat, cultured meat	South-Africa
27	Tosun et al., 2020 [22]	content analysis	-	meat substitutes in general (but mostly plant-based protein)	Turkey
28	Van Loo et al. (2020) [29]	discrete choice experiment	1 830	meat burger (beef), plant-based (heme-protein and pea protein), cultured meat	USA

Notes: The names in the "Focus (with the original description)" column follow the description used in the original studies. Source: authors' own collection (2021).
